# Human Papillomavirus in Brazilian women with and without cervical lesions

**DOI:** 10.1186/1743-422X-8-4

**Published:** 2011-01-05

**Authors:** Michelle Oliveira-Silva, Camila X Lordello, Lucília MG Zardo, Cibele R Bonvicino, Miguel AM Moreira

**Affiliations:** 1 Instituto Oswaldo Cruz, Rio de Janeiro, Brazil; 2 Universidade Federal do Rio de Janeiro, Brazil; 3 Genetics Division, Instituto Nacional de Câncer, Rio de Janeiro, Brazil; 4 Integrated Service Tecnology in Cytology, Instituto Nacional de Câncer, Rio de Janeiro, Brazil

## Abstract

**Background:**

Human Papillomavirus (HPV) high-risk (HR) types are the causal factor for cervical cancer and premalignant dysplasia. Data on frequency of HPV types provide a basis to design and evaluate HPV prevention programs. Taking into account the heterogeneity of HPV types across and within populations this study aims to access the HPV frequency in Brazilian women.

**Results:**

We identified 24 different types of HPV, including a *Betapapillomavirus *and a likely new type, previously reported, from 132 women positive for the virus analysed by Hybrid Capture II assay. These women were infected by a single or multiple HPV types and 142 HPV strains were identified. HR types were found in 75% of women and HPV types 16, 18, 45, 58, and 66 had the highest frequency. Significant differences in frequency of HR HPV types were found for presence of cervical lesions, and for different HPV species and women age.

**Conclusions:**

Compared with previous studies in Brazil, our data indicated differences in frequency and HPV type diversity, a significant association of other HR-types but HPV16 and 18 and cervical lesions, and a trend for distinct distribution of HPV types by age.

## Background

Cervical cancer accounts for the third highest mortality amongst cancers in women worldwide, with a higher incidence and frequency in underdeveloped and developing countries [1]. The etiology of cervical cancer, attributed to the high-risk types (HR) of Human Papillomavirus (HPV), has been well established by experimental and epidemiological studies [2-4]. Due to the discovery of more than 100 HPV types and the association of some types with cancer, pre-cancerous lesions and genital warts [5], a series of assays based on Polymerase Chain Reaction (PCR) amplification and nucleic acid hybridization were designed for HPV detection. HPV16 and HPV18 are the most types reported, accounting for approximately 70% of all cervical cancers [6] and are also frequent in women lacking cytological abnormalities in different continents [7,8].

The high frequency of HPV16 and HPV18 in cervical cancer and pre-cancerous lesions lead to development of vaccines against L1 viral capsid proteins of these types [9,10]. However, the distribution and prevalence of HR-HPV types have been shown to vary among populations worldwide [7,11-13] and also in Brazil [14-24], where most of studies were performed in Southeast region, employing different methodologies for HPV detection and typing showing, particularly for HPV18, the largest variation in prevalence [25]. Considering the use of different methodologies for HPV typing, the DNA sequencing is the only procedure capable to recognize all HPV types and variants present in a biological specimen. Despite of direct sequencing is not adequate for the identification of multiple infections, preferentially detecting types over-represented in a sample [26], this method has been used in many studies on HPV prevalence [27-30].

Taking in account that the characterization of HPV types will be valuable to implement immunization polices and to monitor the presence of different HPV types, the present study aim to accesses the diversity of HPV types in women from communities of low socioeconomic status of the Metropolitan region of the city of Rio de Janeiro city, Brazil.

## Methods

### Study Subjects

We studied women from Duque de Caxias and Nova Iguaçu, two municipalities with low socioeconomic status in the state of Rio de Janeiro, Brazil, assisted by the governmental Family Health Program. They had been visited by trained health care professionals and invited to participate in studies for evaluating the efficacy of different methods for detecting cervical lesions [31], and the Hybrid Capture II (HCII) assay for early detection of cervical cancer [32], and also the quality of records on cervical cancer in Brazil [33]. Socio-demographic, cytological data and endocervical samples of these women, collected between December 2001 and July 2002, were used in the present study. Pap tests had not been carried out in any of these women in the last three years before sample collections; they had not been pregnant, had not given birth at least six months before inclusion, have had sexual relation, had not gone through hysterectomy, and were between 25 and 59 years of age. Endocervical samples were obtained using a conical-shaped brush and stored at -20°C in Digene Specimen Transport Medium™ under denaturing conditions. In this present study, only HPV+ women diagnosed previously by HCII assay were analyzed, totalizing 297 women. This study was approved by the Ethics Committee of the Instituto Nacional de Câncer (registration number 19/05).

The conventional cytology results was classified according to the recommendations of Brazilian Ministry of Health and Brazilian Society of Cytology [26], which is based on Bethesda's definition [34].

### Extraction, Amplification and HPV DNA Typing

Samples were submitted to pH neutralization step with addition of HCl 1N. DNA isolation was carried out with QIAamp DNA Mini and Blood Kit (QIAGEN, Helden, Germany) following the manufacturer's instructions, modified at the elution step that was performed with 30 μL of AE buffer.

HPV DNA amplification was performed by nested-PCR with MY09/11 [35] and GP05/06+ [36] primers, the amplicons were purified with the Illustra GFX PCR and Gel Band Purification Kit (GE Healthcare, UK) before being submitted to direct sequencing, using Big Dye Terminator Kit V3.1 (Applied Biosystems), in a ABI 3730 sequencer at the Genomic DNA Sequencing Platform (PDTIS) of FIOCRUZ [37]. The samples that could not be typed by direct sequencing due to overlap of sequence-peaks were cloned with pMOSBlue Blunt Ended kit (GE Healthcare, UK) and eight clones were sequenced for each patient.

Identification of HPV types was carried out with the Blast software http://blast.ncbi.nlm.nih.gov/Blast.cgi and by phylogenetic analysis within the MEGA 4.0 software [38] applying Neighbor-Joining and Kimura's-2-Parameter (K2P) distance model. Phylogenetic analysis included reference sequences from *Alphapapillomavirus*. Sequences from *Betapapillomavirus *and *Deltapapillomavirus *were used as outgroups. The strength of each node was evaluated by bootstrap test with 1,000 replicates. HPV types were epidemiologically and phylogenetically classified following Muñoz et al [5] and de Villiers et al [39], respectively.

### Statistical Analysis

Association between HPV types and cytology results for women with single infection was performed with the χ^2 ^test. Mann-Whitney and Kruskal-Wallis tests were used to analyze differences between age at diagnoses and HPV type for all women.

## Results

A total of 297 women positive for HCII assay had samples available for DNA isolation and 132 of these had HPV DNA successfully amplified. Despite this, there were no significant differences in respect to the cytological results (ASCUS, AGUS, LSIL and HSIL) and age between women that had HPV DNA successfully amplified and those that not had. The mean age of the 132 women were 39.5 years, ranging from 25 to 59 years of age.

A total of 123 women had the HPV type identified totalizing 142 HPV sequences corresponding to women infected with single, multiple HPV types or by different strains of the same type (GenBank accession numbers HQ834551 - HQ834692). Infections by multiple HPV types or by different strains were found among the 39 women that could not be typed by direct sequencing due to overlap of sequence-peaks and were submitted to molecular cloning and clone sequencing. HPV typing carried out with Blast and confirmed by phylogenetic analysis showed the presence of 24 different HPV types, including HPV17, a *Betapapillomavirus *often identified in cutaneous lesions [39], and a new likely type previously reported as SW1 [40]. One hundred and twelve women were found to be infected by a single HPV type and 11 showed co-infection, 9 of which by two types and two by three types. Among 132 women that had the HPV type amplified, 63.6% (84/132) had no cervical lesions, 14.4% (19/132) had atypical squamous cells of undetermined significance (ASCUS) or atypical glandular cells of undetermined significance (AGUS), 9.8% (13/132) had low-grade squamous intraephitelial lesion (LSIL) and 19.7% (26/132) had high-grade squamous intraephitelial lesion (HSIL) (Table 1).

**Table 1:** HPV type and cytological results of the 132 HPV+ women.

	Cytological results*							
		
		HPV type	Normal	ASCUS	AGUS	LSIL	HSIL	No. women
**Single Infection** **(N = 112)**	**High-Risk**	16	19	3	1	1	6	29
		18	13	1	1	0	1	15
		31	3	0	0	1	1	5
		33	4	0	0	0	0	4
		35	1	0	0	0	2	3
		39	1	0	0	1	0	2
		45	4	0	0	1	4	9
		53	2	0	0	0	2	4
		58	5	1	2	2	2	9
		66	4	2	0	0	2	8
		
	**Low-Risk**	6	5	0	0	1	1	7
		43	0	1	1	0	1	2
		
	**Unknown-**	17	0	0	0	1	0	1
	**Risk**	70	4	1	0	0	1	6
		74	1	0	0	1	0	1
		82	1	0	0	0	0	1
		83	0	1	0	3	1	3
		89	1	0	0	0	0	1
		90	1	0	0	0	0	1
		SW1	1	0	0	0	0	1

**Multiple Infection** **(N = 11)**	**Double**	16, 66	1	0	0	0	0	1
		30, 35	0	1	0	0	0	1
		16, 83	1	1	1	0	0	2
		18, 83	1	0	0	0	0	1
		16, 18	1	0	0	0	1	2
		18, 72	1	0	0	0	0	1
		16, 45	1	0	0	0	0	1
		
	**Triple**	16, 52, 83	0	0	0	0	1	1
		6, 16, 56	1	0	0	0	0	1

**HPV type not identified**			7	1	0	1	0	9

**Total**								**132**

*ASCUS, Atypical squamous cells of undetermined significance; AGUS, Atypical glandular cells of undetermined significance; LSIL, Low-grade squamous intraephitelial lesion; HSIL, High-grade squamous intraephitelial lesion.

The frequency of HPV HR-types among HPV+ women was 75% (99/132 women), with a highest frequency for HPV16 (28%; 37/132), followed by HPV18 (14.4%; 19/132), HPV45 (7.6%; 10/132), HPV58 (6.8%; 9/132), HPV66 (6.8%; 9/132), HPV31 (3.8%; 5/132) and HPV33 (3.0%; 4/132). Considering only the 84 HPV+ women with normal cytology, we found frequencies of 28.6% (24/84) for HPV16 and 19.0% (16/84) for HPV18.

**Figure 1:**
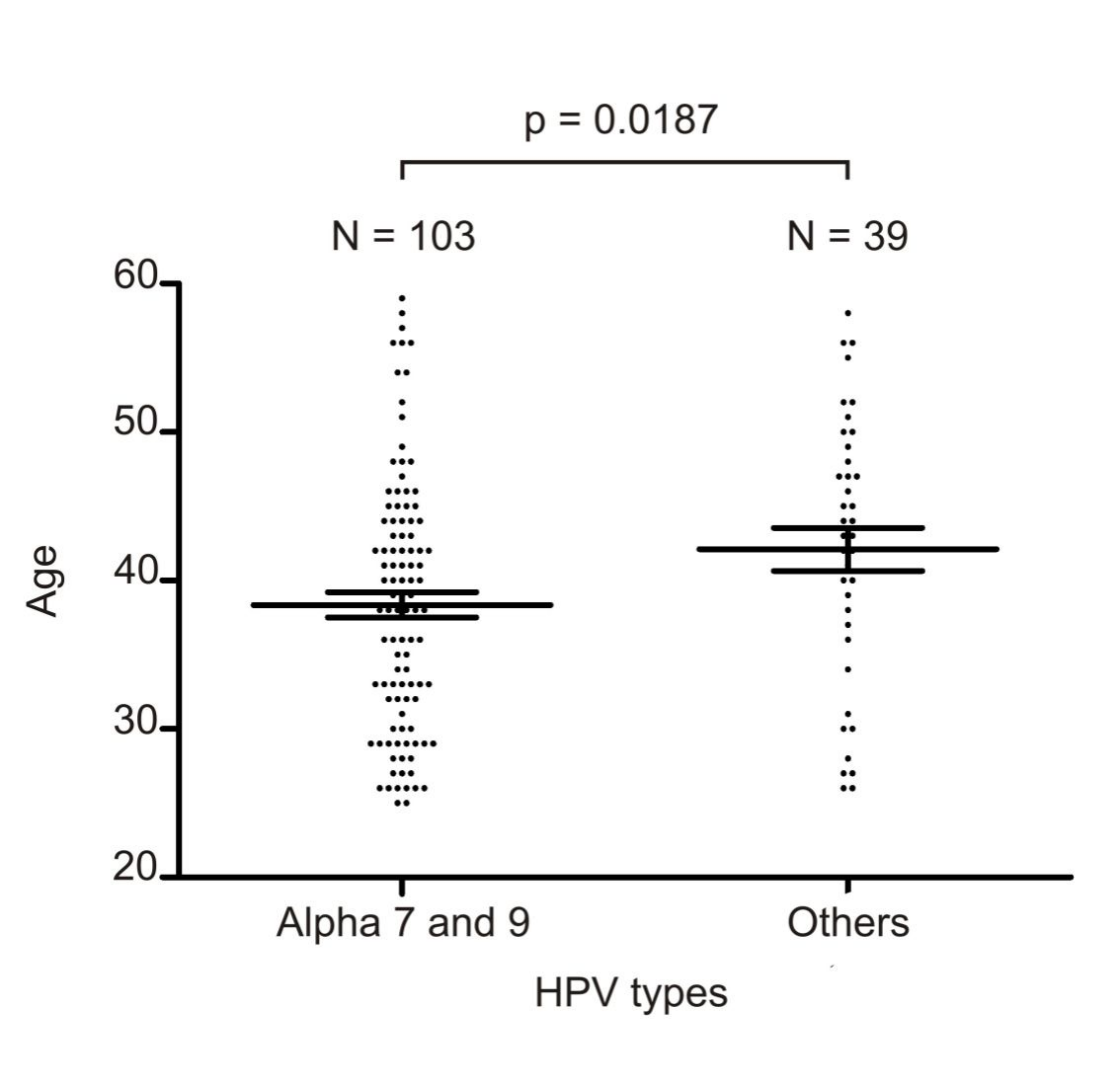
**HPV types and age**. Comparison of the distribution of infections by HPV alpha-7 and 9 versus other HPV species and women age. N = Number of HPV strains identified considering single and multiple infections. Bars indicate the mean and standard error of the mean.

A significant lower proportion of LSIL and HSIL was found among women infected by HPV16 and/or HPV18 when compared to the ones infected by other HR-types (χ^2 ^test, p = 0.0411). Our data also showed that infection by alpha-7 (including HPV18, 39, 45, 59, 68 and 70) and alpha-9 species (including HPV16, 31, 33, 35, 52, 58 and 67) presented a significant distinct distribution by age at diagnosis respective to women positive for other HPV types (Mann-Whitney test, p = 0.0187) (Figure 1). However, separate comparisons among alpha-7 infections, alpha-9 infection, and infections by other HPV types, did not show a significant different distribution by age (Kruskal-Wallis test, p = 0.06).

## Discussion and Conclusions

All cervical samples included in the present study were HPV+ by the HCII assay, which include probes for detection of 18 *Alphapapillomavirus *types (HR types: HPV16, 18, 31, 33, 35, 39, 45, 51, 52, 56, 58, 59 and 68; LR types: HPV6, 11, 42, 43 and 44). However, we identified 12 HPV types (HPV17, 30, 53, 66, 70, 72, 74, 82, 83, 89, 90 and SW1) not included in that set of probes. These findings reinforced previous evidence of cross-hybridizations among probes used in HCII test [41-45]. Interestingly, the *Betapapillomavirus *HPV17 was also identified, a type frequently associated to cutaneous lesions, indicating that probes contained in the HCII test were capable of cross-hybridizing with viruses not belonging to *Alphapapillomavirus*.

In Brazil, most studies on HPV frequency used as inclusion criteria the suspicion of HPV infection, presence of cervical lesions or cancer. In our study, these criteria were not used, a reason why we compared our findings with studies with similar inclusion criteria [15,19-24]. Three of these studies were performed in Northeast region, two in the same city (Recife), and the HPV frequency reported were discrepant among them and also in comparison with our study. Franco et al [21], carried out a study with 122 HPV+ in João Pessoa city, using dot blot hybridization method for typing and found a lower frequency of HPV45 (3.1%) and higher frequency of HPV33 (13.5%) than here reported (7.6% and 3.0%, respectively). In Recife city, Lorenzato et al [22] analyzing 214 HPV+ women and using PCR/RFLP for HPV typing, found a higher frequency of HPV31 (21.4%) and a lower frequency of HPV18 (2.4%) in comparison with our findings (3.8% and 14.4%, respectively). The third study by Baldez et al [20], also conducted in Recife, analyzed 213 HPV+ women using specific primers for PCR amplification of four HPV types and found a higher frequency of HPV16 (78%) and HPV31 (15.5%), and lower frequency of HPV18 (2.8%) in respect to our data (28.0% of HPV16; 3.8% of HPV31 and 14.4% of HPV18).

In a study performed in Metropolitan region of Rio de Janeiro city at the Southeast region of Brazil, Oliveira et al [19] analyzing 82 HPV+ young women (between 14 to 26 years old), using PCR/RFLP for HPV typing, reported a higher frequency of HPV31 (12.2%) than the one found by us (3.8%), and accounting for the second most frequent type after HPV16. In addition, a lower frequency for HPV16 (18.3%) and HPV18 (2.4%) were observed in comparison to our data (28.0% and 14.4%, respectively). In state of São Paulo, also in Southeast region of Brazil, Lippman et al [15], analyzed 135 HPV+ women of 18 to 40 years of age, and employing PCR/RFLP for HPV typing, detected a large diversity of HPV types with lower frequencies for HPV16 (17%), HPV45 (2.2%), HPV58 (4.4%) and HPV66 (2.2%) in comparison to our data (28.0% for HPV16; 7.6% for HPV45; 6.8% for HPV58 and 6.8% for HPV66).

In two studies performed at the South region of Brazil, the first by Krambeck et al [24] in the state of Santa Catarina, using PCR/RFLP for HPV typing, in 29 HPV+ women, and the second by Rosa et al [23] in the state of Rio Grande do Sul, using specific primers for typing HPV16, HPV18, and HPV31, in 179 HPV+ women, reported lower frequencies for HPV16 (17.2% and 18.6%, respectively) than the found here (28.0%). However, the second most frequent types identified in these studies (HPV53 with 10.3% and HPV31 with 15.8%, respectively) were found with higher frequencies than in our study (HPV53 with 3.0% and HPV31 with 3.8%). Furthermore, the HPV18 was not reported in state of Santa Catarina although this type has been found in the state of Rio Grande do Sul with lower frequency (3.3%) than the observed by us (14.4%).

Concerning the 84 HPV+ women with normal cytology, we found a higher frequency of HPV16 (28.6%; 24/84) and HPV18 (19.0%; 16/84) than in a meta-analysis, restricted to women with normal cytology, carried out for South America [7] with 15% and 5%, respectively. In addition, this meta-analysis found a frequency of 7% for HPV58, the second most frequent type, similarly to our sample (6.0%; 5/84) in which this type was the fourth most frequent. These data provide a complementary picture to studies of HPV type distribution in women with cancer or precancerous lesions.

Our results indicated a trend for a higher proportion of lesions in women infected by HR-types other than HPV16 and/or HPV18, indicating that other HR-HPVs must also be considered for further implement appropriate immunization and monitoring policies. Moreover, the considerable difference in frequency of HPV types amongst previous studies (*e.g*.: ranging from 17.2% to 78.7% for HPV16, and from 0% to 14.4% for HPV18, among HPV+ women), evidences the need to further investigations to improve information of geographical distribution of HPV types in Brazil using standardized methodologies to HPV detection and typing.

## Abbreviations

AGUS: Atypical glandular cells of undetermined significance; ASCUS: Atypical squamous cells of undetermined significance; HCII: Hybrid Capture II assay; HPV: Human Papillomavirus; HPV HR: Human Papillomavirus of High-Risk for cancer; HPV LR: Human Papillomavirus of Low-Risk for cancer; HSIL: High-grade squamous intraephitelial lesion; LSIL: Low-grade squamous intraephitelial lesion; PCR: Polymerase Chain Reaction; RFLP: Restriction Fragment Length Polymorphism

## Competing interests

The authors declare that they have no competing interests.

## Authors' contributions

MOS and CXL contributed to conception and design, acquisition, analysis and interpretation of data. CXL and MOS performed the molecular procedures, phylogenetic analyses, and drafted the manuscript. CRB revised the data and contributed with important intellectual content. MAMM and LMGZ conceived participated in study design and coordination, and helped to draft the manuscript. All authors read and approved the final manuscript.
